# FAH Domain Containing Protein 1 (FAHD-1) Is Required for Mitochondrial Function and Locomotion Activity in *C*. *elegans*

**DOI:** 10.1371/journal.pone.0134161

**Published:** 2015-08-12

**Authors:** Andrea Taferner, Haymo Pircher, Rafal Koziel, Susanne von Grafenstein, Giorgia Baraldo, Konstantinos Palikaras, Klaus R. Liedl, Nektarios Tavernarakis, Pidder Jansen-Dürr

**Affiliations:** 1 Institute for Biomedical Aging Research and Center for Molecular Biosciences Innsbruck (CMBI), Universität Innsbruck, Rennweg 10, 6020, Innsbruck, Austria; 2 Institute for General, Inorganic and Theoretical Chemistry and Center for Molecular Biosciences Innsbruck (CMBI), Universität Innsbruck, Innrain 80–82, 6020, Innsbruck, Austria; 3 Institute of Molecular Biology and Biotechnology, Foundation for Research and Technology-Hellas, Heraklion, Crete, Greece; 4 Department of Basic Sciences, Faculty of Medicine, University of Crete, Heraklion, Crete, Greece; CSIR-Central Drug Research Institute, INDIA

## Abstract

The fumarylacetoacetate hydrolase (FAH) protein superfamily of metabolic enzymes comprises a diverse set of enzymatic functions, including ß-diketone hydrolases, decarboxylases, and isomerases. Of note, the FAH superfamily includes many prokaryotic members with very distinct functions that lack homologs in eukaryotes. A prokaryotic member of the FAH superfamily, referred to as Cg1458, was shown to encode a soluble oxaloacetate decarboxylase (ODx). Based on sequence homologies to Cg1458, we recently identified human FAH domain containing protein-1 (FAHD1) as the first eukaryotic oxaloacetate decarboxylase. The physiological functions of ODx in eukaryotes remain unclear. Here we have probed the function of *fahd-1*, the nematode homolog of FAHD1, in the context of an intact organism. We found that mutation of *fahd-1* resulted in reduced brood size, a deregulation of the egg laying process and a severe locomotion deficit, characterized by a reduced frequency of body bends, reduced exploratory movements and reduced performance in an endurance exercise test. Notably, mitochondrial function was altered in the *fahd-1(tm5005)* mutant strain, as shown by a reduction of mitochondrial membrane potential and a reduced oxygen consumption of *fahd-1(tm5005)* animals. Mitochondrial dysfunction was accompanied by lifespan extension in worms grown at elevated temperature; however, unlike in mutant worms with a defect in the electron transport chain, the mitochondrial unfolded protein response was not upregulated in worms upon inactivation of *fahd-1*. Together these data establish a role of *fahd-1* to maintain mitochondrial function and consequently physical activity in nematodes.

## Introduction

The fumarylacetoacetate hydrolase (FAH) protein superfamily branches out through all organisms, from prokaryotes to humans, comprising a diverse set of enzymatic functions. Its distinguishing feature is the highly conserved catalytic domain structurally characterized by the FAH fold. Although the FAH fold is highly conserved, FAH superfamily members cover a wide range of diverse enzymatic activities, including hydrolases, isomerases, and decarboxylases. Overall, the FAH superfamily includes many prokaryotic members with very distinct functions that lack homologs in eukaryotes. This can be explained by the fact that these enzymes are part of highly specialized metabolic pathways, involving chemical compounds that higher organisms are unable to convert and utilize for their metabolism [1]. One recently identified prokaryotic member of the FAH superfamily found in *Corynebacterium glutamicum*, referred to as Cg1458, was characterized as a novel soluble oxaloacetate decarboxylase (ODx) [2,3]. Whereas eukaryotic ODx enzymes were not identified so far, we recently found that human FAH domain containing protein 1 (FAHD1), a mitochondrial protein [4], displays ODx activity, establishing the first eukaryotic oxaloacetate decarboxylase [5]. The physiological function of ODx enzymes in eukaryotes remains to be established. When the FAHD1 gene was deleted in mice, we found that FAHD1^-/-^ mice were viable [5] and did not display striking phenotypical abnormalities (H. Pircher *et al*., unpublished), suggesting that compensatory mechanisms may exist in mice which may obscure phenotypical changes resulting from deletion of the FAHD1 gene. Here, we identified the nematode homolog of FAHD1, referred to as *fahd-1*, and assessed the consequences of its mutational inactivation in the nematode *C*. *elegans*, a lower eukaryotic model organism widely used for the analysis of metabolic regulation [6] and mitochondrial physiology [7].

## Materials and Methods

### Computational homology modeling of nematode FAHD-1 structure

A homology model was generated to compare the structure of FAHD-1 from *C*. *elegans* based on the sequence of ZK688.3 (NP_498715.1). As expected, sequence search revealed the FAH domain containing structures as suitable templates. Sequence identity is 46.3% for FAHD1 (PDB code 1SAW), with a similarity of 64.5%. Although this structure is the closest in sequence, we selected the FAH protein from *Yersinia pestis* CO92 (PDB code 3S52) as template, having a better resolution and a completely resolved structure for chain A (closed and structured lid). The template and the target sequence share 39.7% sequence identity. The model was generated with MOE homology modeling tool (Chemical Computing Group, MOE release 2013.08) with chain A and D of 3S52 as templates for a dimer model using the force field option Amber12EHT. The model had unexpected cis amid configurations for Arg8 in chain A and B as well as Lys13 in chain A, which are solvent exposed or in the dimer interface respectively. They are associated with outliers in the Ramachandran plot for the neighboring Asn9 in chain B and Lys13. Additionally the distal residue Asn147 in chain B and Pro135 in both subunits have suspicious backbone configurations. However, the binding site shows no parameters indicating quality issues in the model structure. In the active site, side chain orientations of Arg100 and Glu65 were manually adapted. The initial model was complemented by water positions and co-crystallized ions from the structure of human FAHD1 (PDB code 1SAW) and not the template, as the latter does not include the magnesium ion in the active site. Eight individual water molecules forming too close contacts with the model were removed. To allow water and active site adaptions to the magnesium ion, the assembly was energy minimized in several steps with decreasing positional restraints on the atoms.

### Nematode strains and cultivation

*C*. *elegans* were cultured using standard protocols [8,9] at 20°C unless stated otherwise. The wild-type strain N2 ancestral and the transgenic strain SJ4100: N2;*Is*[p_*hsp-6*_GFP] were obtained from the Caenorhabditis Genetics Center (CGC; University of Minnesota, Minneapolis, USA; https://www.cbs.umn.edu/research/resources/cgc). The deletion mutant *fahd-1(tm5005)* was obtained from the ‘National Bioresource Project for the Experimental Animal ‘Nematode *C*. *elegans*” (Head: Dr. Shohei Mitani, Tokyo Women’s Medical University, Tokyo, Japan) and backcrossed six times to N2 ancestral. The reporter lines N2;*Ex*[p_*fahd-1*_GFP; pRF4] and N2;*Ex*[p_*fahd-1*_FAHD-1::GFP; pRF4] were generated in our laboratory (see below).

### Generation of reporter lines

For the transcriptional reporter line N2;*Ex*[p_*fahd-1*_GFP; pRF4], the promoter region of the *fahd-1* gene (approx. 2 kb upstream of the start codon; obtained by PCR using genomic DNA as template) was cloned in front of GFP in the plasmid vector pPD95.75 (gift from Andrew Fire; Addgene plasmid #1494 [10]) using the primers GCG-AAGCTT-ATATCAGGTTCCTCATACCAGG (forward primer; including restriction site for HindIII) and GC-GGATCC-GTCTAAAATGTAGTTTTTTTTGTTTC (reverse primer; including restriction site for BamHI). For the translational reporter line N2;*Ex*[p_*fahd-1*_FAHD-1::GFP; pRF4], the full-length *fahd-1* gene (including promoter region, exons, introns) was cloned in-frame in front of GFP of the same plasmid vector using the primers GCG-AAGCTT-ATATCAGGTTCCTCATACCAGG (forward primer; including restriction site for HindIII) and GC-GGTACC-AC-CTGAACATTAAATTTGGAATTCAA (reverse primer; including restriction site for KpnI). The reporter constructs were injected into the cytoplasm of the syncytial gonad of young adult N2 ancestral *C*. *elegans* following standard protocols [11]. For identification of transformants, pRF4, a plasmid that carries the *rol-6(su1066)* dominant transformation marker, was co-injected. The F1 offspring was observed for evidence of the transgenic marker and each F1 transformant was separately cloned onto a new plate. F2 animals that had inherited and expressed the transgenic array were considered as stable lines.

### RNAi constructs

The RNAi clone for *cco-1* (DFCIp3320A1211012D) was generated in the laboratory of Dr. Marc Vidal and obtained via Source BioScience (http://www.lifesciences.sourcebioscience.com). The *fahd-1* RNAi clone was generated by targeting the entire coding region of the gene using the primers TA-CCATGG-CACCAGCTCTACAGTAGTCA (forward primer; including restriction site for NcoI) and TA-CTCGAG-GGACCATTGGTCTCGTTAGG (reverse primer; including restriction site for XhoI). The PCR product was cloned into the L4440 vector (gift from Andrew Fire; Addgene plasmid #1654; [10]) and transformed into *E*. *coli* HT115(DE3) [12].

### Basic phenotypical analysis

Phenotypical assays were performed using 4-day old worms because many phenotypes are most pronounced at this age. The basic phenotype of *fahd-1* mutant *C*. *elegans* was determined using established protocols [13]. To determine brood size, single worms were put on fresh NGM agar plates seeded with *E*. *coli* OP50 every 12 hours for the entire length of the reproductive phase. The progeny (larvae) were counted after 2 days. The brood size of 10 individual worms of each strain and condition was examined. Egg-laying behavior was analyzed by putting 4-day old worms individually in M9 buffer (50 μl per well in a 96-well plate) and counting eggs outside the worms after 1, 2, 3, and 4 hours. The experiment was performed 3 times; each time 24 worms per strain were analyzed. To test locomotion, 4-day old hermaphrodites were placed on an empty (i.e. without bacterial lawn) NGM agar plate. After letting them adjust for 30 seconds, the body bends (one body bend is deemed as one sinusoidal movement until the worm reaches the same posture again) for one minute. The experiment was performed 3 times; each time 10 individual worms per strain were analyzed. The radial dispersal rate (describing the distance an animal moves away from the starting point in a given time period) was determined as follows: 4-day old hermaphrodites were put in the center of an NGM agar plate seeded with *E*. *coli* OP50. The radial dispersal was determined after 30 seconds, 1 minute, and 2 minutes. The agar plate was radially scaled in different zones; zone 1 was 0–5 mm, zone 2 was 5–15 mm, zone 3 was 15–25 mm and zone 4 was 25–35 mm from the starting point. The experiment was performed 3 times; each time between 20 and 50 worms per strain were analyzed. Swimming endurance performance was tested by putting 4-day old animals in M9 buffer (50 μl per well in a 96-well plate) and screening every hour for characteristic thrashing behavior. Worms that did not show these characteristic thrashing movements were considered as ‘not swimming’. The experiment was performed 3 times; each time 24 worms per strain were analyzed.

### Analysis of mitochondrial parameters

Analysis of mitochondrial parameters was performed with 3-day old adults to avoid excessive contamination with eggs and progeny. The mitochondrial membrane potential was examined using the membrane potential-sensitive dye tetramethylrhodamine, ethyl ester (TMRE; obtained from Life Technologies, Carlsbad, USA). Approx. 56-hours old animals were placed on NGM agar plates containing 100 nM TMRE, stained overnight and pictured when they were 72 hours old using epifluorescence microscopy. The quantification of the fluorescence signal (pixel intensity) was performed using ImageJ (imagej.nih.gov/ij/). The fluorescence signal of 30 individual animals per strain was determined. The oxygen consumption rate was measured using an OROBOROS Oxygraph 2K (Oroboros Instruments GmbH, Innsbruck, Austria) as described [14], with the following modifications: Approx. 300 3-day old worms were collected in S basal buffer, washed and delivered into the chamber in 2.5 ml of S basal medium. The chamber was kept at 20°C and measurements were performed for 15 min. Obtained data were analyzed using DatLab 4 software (Oroboros Instruments GmbH, Innsbruck, Austria). The slope of the straight portion of the plot was used to derive the oxygen consumption rate. Worms were recovered after respiration measurements and counted. Respiration rates were normalized to number of worms. The experiment was performed 4 times. Mitochondrial localization was determined by co-staining the translational reporter N2;*Ex*[p_*fahd-1*_FAHD-1::GFP; pRF4] with MitoTracker Red (Life Technologies, Carlsbad, USA). 3-day old animals were stained overnight with 100 nM MitoTracker Red that was applied to the NGM agar plates and pictured when they were 4 days old.

### Lifespan determination

Lifespan experiments were carried out following standard protocols [15] at 20°C and/or 25°C. To tightly synchronize the animals used in the lifespan assays, adult hermaphrodites were allowed to lay eggs for 2 h on NGM plates seeded with *E*. *coli* OP50. The synchronized population was allowed to develop under defined conditions. At L4 larval stage animals were transferred to fresh plates in groups of 15–20 worms per plate for a total of 200 worms per experiment. The day the eggs were laid was considered as t = 0. Animals were transferred to fresh plates every day during their reproductive period to separate them from their progeny and every second day thereafter until the end of their lifespan. The worms were checked every day for touch-provoked movement and pharyngeal pumping. When no reaction could be observed, they were considered as dead. Worms that died due to internally hatched eggs, an extruded gonad or intestine, or desiccation due to crawling off the plates, or worms that buried themselves in the agar and could not be transferred to fresh plates were censored and not incorporated into the analysis. Each lifespan assay was carried out at least three times. The software GraphPad Prism 5 (GraphPad Software, Inc., La Jolla, USA) was used for statistical analysis. Survival curves were created using the product-limit method of Kaplan and Meier. The log-rank (Mantel-Cox) test was used to evaluate differences between survivals and determine the corresponding p values. For RNAi lifespan experiments, worms were placed on NGM plates containing 1 mM IPTG and 50 μg/ml ampicillin and seeded with *E*. *coli* HT115(DE3) bacteria transformed with either the test RNAi construct or the empty L4440 vector (gift from Andrew Fire; Addgene plasmid #1654) as control. In order to abolish all maternally inherited mRNAs, the second generation grown on RNAi bacteria was used for lifespan experiments.

### Western blotting

To avoid contamination of protein lysates with bacterial proteins, freshly starved worms were washed off the agar plate with M9 buffer, washed several times and then snap frozen in liquid nitrogen. The worm pellets were thawed on ice and 150 μl of protein lysis buffer (50 mM Tris-HCl pH 8.0, 150 mM NaCl, 1% NP-40, 0.5% DOC, 0.1% SDS; supplemented with protease inhibitors (1 Complete Mini EDTA-free tablet per 10 ml, Roche Diagnostics GmbH, Mannheim, Germany)) were added. The worms were broken up using a Branson Sonifier 250 and then incubated on ice for 30 min. The lysate was cleared by centrifugation for 20 min at 16,000 x g and 4°C. The protein concentration was determined using Bradford assay. Lysates (30 μg total protein) were separated by SDS-PAGE and blotted onto a PVDF membrane. Peptide-specific rabbit polyclonal antibody against FAHD-1 (2 μg/ml; generated by BioGenes GmbH, Berlin, Germany and purified in our lab), and anti-rabbit HRP-conjugated secondary antibody (Dako P0399, 1:2,500, Dako, Glostrup, Denmark) were applied by standard Western blot protocol. β-actin antibody JLA20 (Calbiochem CP01, 1:10,000, Calbiochem, La Jolla, USA) was used for loading control with an anti-mouse HRP-conjugated secondary antibody (Dako P0447, 1:10,000). Detection was achieved by ECL Prime (GE Healthcare, Chalfont St Giles, UK).

### Single worm duplex PCR

The genotype of the *fahd-1(tm5005)* animals was determined by single-worm duplex PCR using standard protocols [16]. Three primers (first forward primer (F1; in front of deletion): CATGTCTTCACTCGCTGGAT; second forward primer (F2; within deletion): CATTGGAGGTTACACTGTCG; reverse primer (R1): GATCATCACATCGGTACGAC) that produced two differently sized products (one that arises from the wild-type allele and one that arises from the mutant allele) were used. Primers F1 and R1 produced a 337 bp amplicon that was specific to the mutant allele. Primers F2 and R1 produced a 220 bp amplicon that was specific to the wild-type allele. The product of primers F1 and R1 of the wild-type allele was too long (573 bp) to be significantly amplified. The PCR products were analyzed by agarose gel electrophoresis.

### Fluorescence microscopy

*C*. *elegans* were mounted in a drop of M9 buffer containing 10 mM NaN_3_ or 10 mM levamisole (levamisole hydrochloride, Sigma, St. Louis, USA) as anesthetics on glass microscope slides. Fluorescent images were taken using a Nikon Eclipse TE300 connected to a Nikon Digital Sight DS-U2 camera. Confocal images were taken using an AxioObserver Z1 LSM710 confocal microscope and a Zeiss Axiophot equipped with the BioRad μRadiance Confocal Scanning System.

### Statistical analysis

Each experiment was performed at least three times (see figure legends and individual methods for details). Mean value and standard deviation were calculated. The significance levels were determined using the Student’s t-test unless stated otherwise (see Lifespan determination). p values <0.05 were considered as significant.

## Results

### Identification of *fahd-1*, the worm homolog of human FAHD1

**Fig 1:**
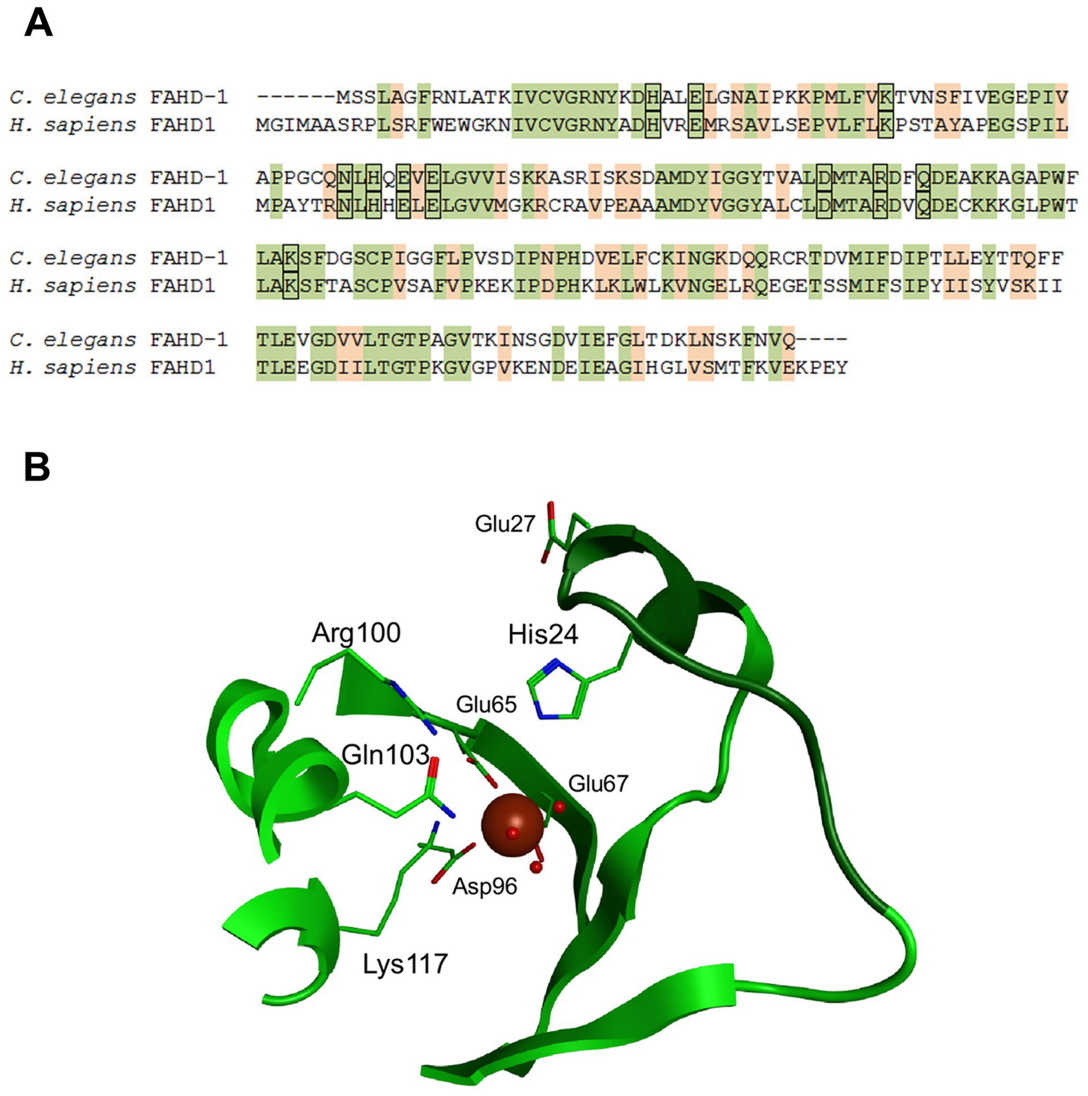
ZK688.3 is the nematode homolog of FAHD1. (A) The hypothetical protein ZK688.3 (NP_498715.1; now FAHD-1) was identified as the only *C*. *elegans* ortholog of human FAHD1 by BLAST search (E value of 9e-71; http://blast.ncbi.nlm.nih.gov/), and confirmed by reverse BLAST. A comparison of the amino acid sequences revealed 47% identity between the two proteins. Amino acids relevant for the ODx function of FAHD1 are boxed. (B) Conserved residues (shown as sticks) arrange as known from FAHD1 or Cg1458. The central magnesium ion (brown sphere) is coordinated by the negatively charged Glu65, Glu67 and Asp96. Around the catalytic site typical fold elements of the protein backbone are shown as ribbon.

To identify the *C*. *elegans* homolog of the human oxaloacetate decarboxylase (ODx) FAHD1 [5], a bioinformatics search was carried out. The gene with the provisional name ZK688.3 was identified as the closest and only homolog of FAHD1 in the nematode genome. This annotation was also confirmed by reverse BLAST (data not shown). The nematode protein, referred to as FAHD-1, contains 48% identical amino acids relative to human FAHD1 and shows high amino acid sequence similarity in the presumed catalytic center (Fig 1A); in particular, all of the amino acids which are presumed to be relevant for ODx function in human FAHD1 [5] are conserved in the nematode homolog. Moreover, computational modeling revealed a structure of the catalytic center highly similar to human FAHD1 (Fig 1B).

### Analysis of *fahd-1* expression in nematodes

**Fig 2:**
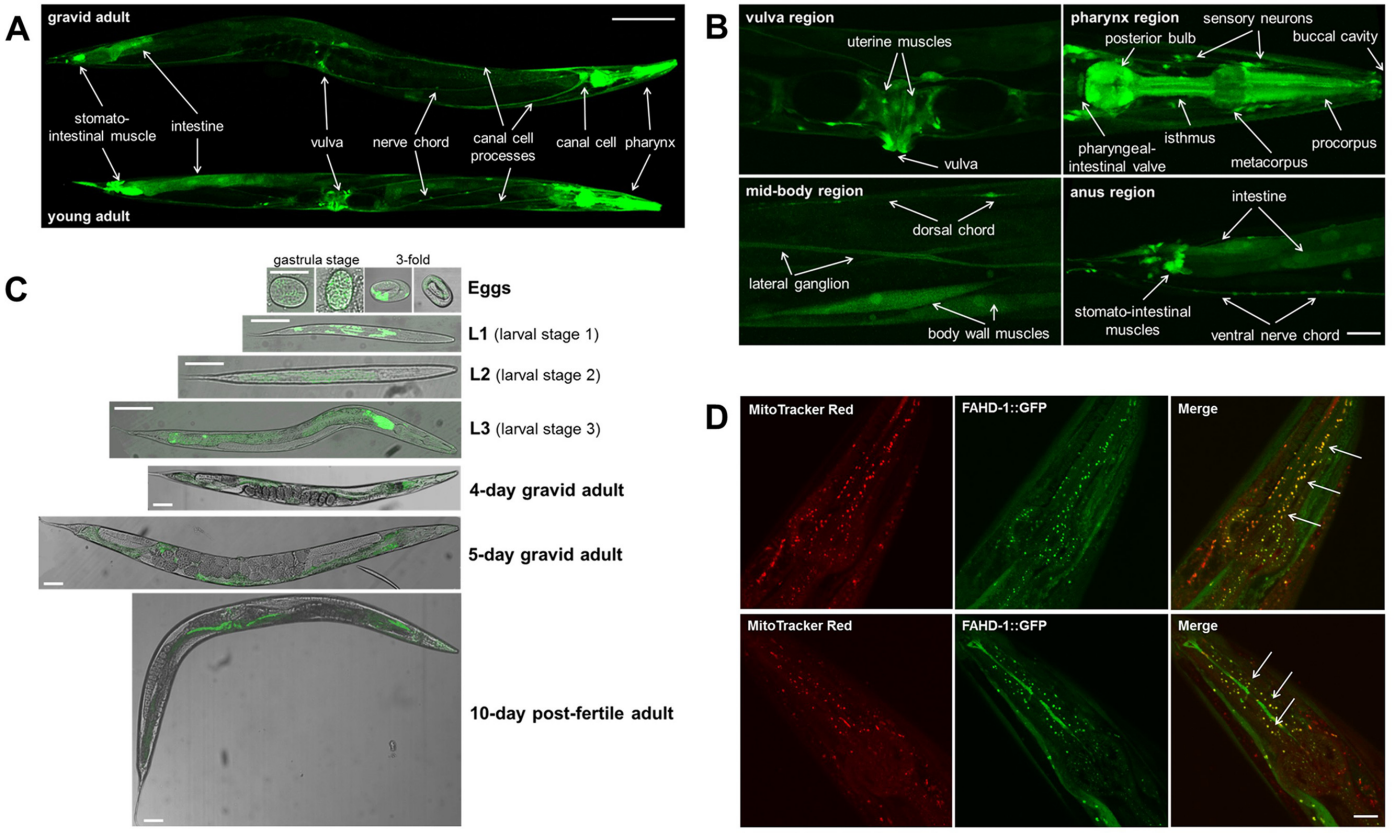
Expression and subcellular localization of FAHD-1 in nematodes. (A) Young adult and gravid adult hermaphrodites of the transcriptional reporter N2;*Ex*[p_*fahd-1*_GFP, pRF4] were monitored by confocal microscopy. *fahd-1* seems to be expressed in most tissues, including pharynx, canal cell, neurons, vulva, intestine, and stomato-intestinal muscle. Representative pictures are shown. Size bar = 100 μm. (B) Confocal images of young adult *C*. *elegans* of the transcriptional reporter N2;*Ex*[p_*fahd-1*_GFP, pRF4]. Representative images are shown. Size bar = 10 μm. *Upper left panel*: Detailed picture of *fahd-1* expression in the peri-vulvar region; vulva and uterine muscles are stained positive. *Upper right panel*: Detailed picture of *fahd-1* expression in the pharyngeal region; buccal cavity, procorpus, metacorpus, isthmus, posterior bulb, and pharyngeal-intestinal valve, as well as sensory neurons in the head were stained positive. *Lower left panel*: Detailed picture of *fahd-1* expression in the mid-body region; body wall muscles, lateral ganglion, dorsal chord (DC), and ventral nerve chord (VNC) were stained positive. *Lower right panel*: Detailed picture of *fahd-1* expression in the anus region; the stomato-intestinal muscles, the anus and the intestine were stained positive. (C) Temporal expression pattern of *fahd-1*. The translational reporter N2;*Ex*[p_*fahd-1*_FAHD-1::GFP, pRF4] was examined (and pictured) by confocal fluorescence microscopy from the time point the eggs were laid at gastrula stage to 10-day old post fertile adults. GFP fluorescence was overlaid with phase-contrast microscopy. The FAHD1-1::GFP fusion protein is present in all stages from gastrulation to old worms. Representative images are shown. Size bar = 50 μm. (D) Young adult hermaphrodites of the translational reporter N2;*Ex*[p_*fahd-1*_FAHD-1::GFP, pRF4] expressing the FAHD-1::GFP fusion protein (shown in green) were stained with 100 nM MitoTracker Red (shown in red) overnight. Shown here are confocal pictures of the pharynx region of the worm recorded in the green and red channel, as well as the merged pictures. Regions of overlapping staining (shown in yellow) are indicated by arrows. Representative pictures are shown. Size bar = 10 μm.

To analyze the expression pattern of FAHD-1 in adult worms, a transcriptional reporter was generated, in which a GFP reporter gene was driven by the *fahd-1* promoter. After injection of the reporter gene, expression was monitored by fluorescence microscopy. We found a broad expression of FAHD-1 in various tissues of adult worms (Fig 2A), including the body wall muscles, the vulva, the pharynx as well as various types of neurons and the canal cell (Fig 2B).

To determine both the time course of expression and the subcellular localization of the protein, a translational reporter was constructed in which expression of a FAHD-1::GFP fusion protein is driven by the *fahd-1* promoter. This reporter strain was used to monitor expression of the gene during development and adult life (Fig 2C). We found that *fahd-1* is expressed from the early gastrula stage throughout the adult life in various tissues. We also used the translational reporter to monitor subcellular localization of FAHD-1 in worms co-stained with MitoTracker Red. As shown in Fig 2D, the signals obtained by FAHD-1::GFP and MitoTracker Red were largely colocalized, indicating specific mitochondrial localization of FAHD-1 in nematodes, consistent with previous findings that human FAHD1 localizes to mitochondria [4].

### Characterization of mutant strain *fahd-1(tm5005)*

**Fig 3:**
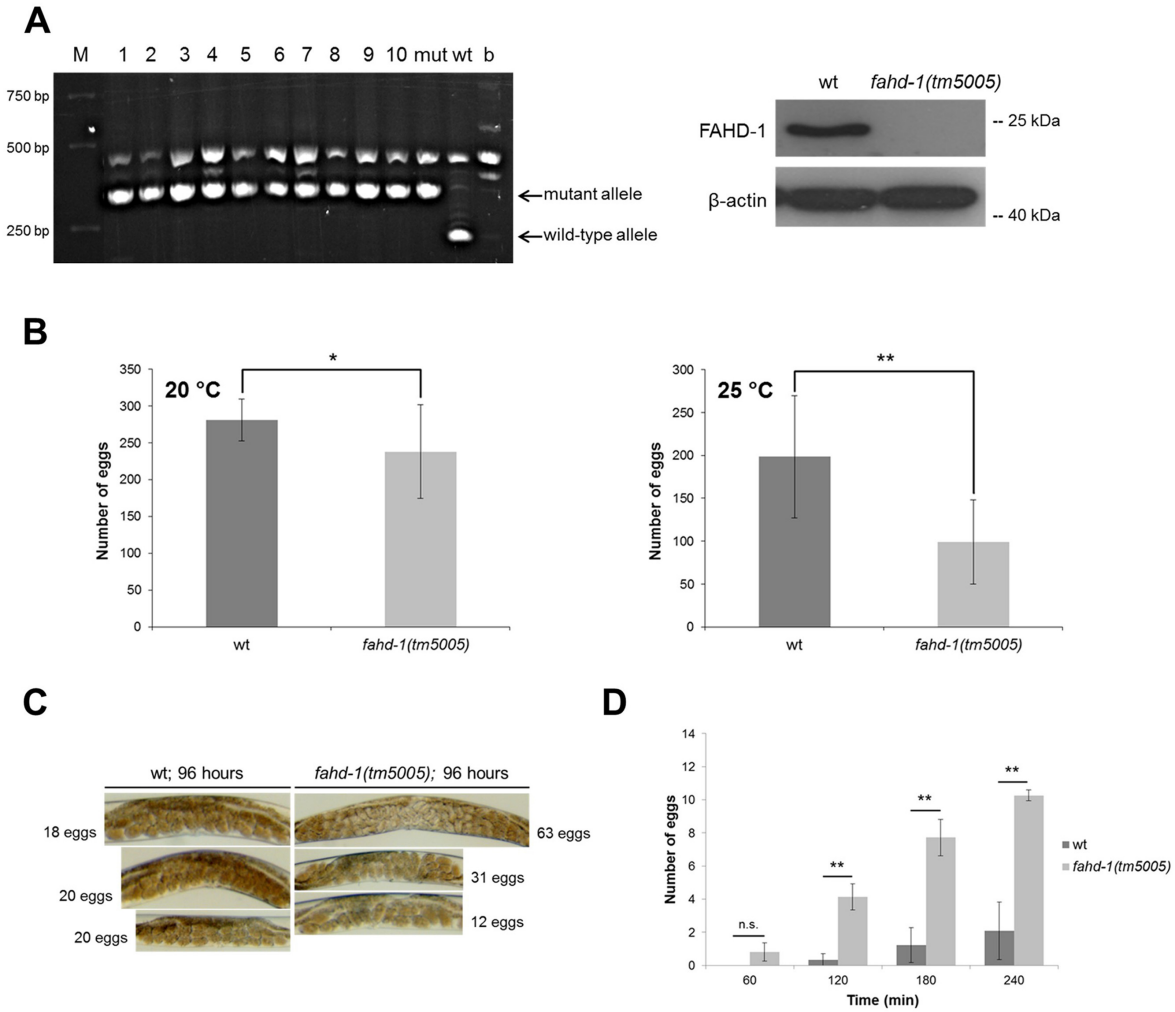
Characterization of *fahd-1* deletion mutant animals. (A) *Left panel*: Single worm duplex PCR was performed on ten animals of the *fahd-1(tm5005)* mutant strain, to confirm the deletion on DNA level. Three primers were used, generating PCR products of 220 bp for the wild-type allele and 337 bp for the mutant allele. M = size marker; 1–10 = 10 different backcrossed *fahd-1(tm5005) C*. *elegans*; mut = mutant allele; wt = wild-type allele; b = *E*. *coli* OP50 (*C*. *elegans* bacterial food; to identify bands derived from bacterial contamination). *Right panel*: To confirm the deletion of the protein, protein lysates were prepared from wild-type and *fahd-1(tm5005)* mutant animals, as indicated. FAHD-1 protein was detected by Western blot using a peptide-specific rabbit polyclonal antibody against FAHD-1. β-actin served as a loading control. (B) Overall brood size was compared by counting progeny in wild-type and *fahd-1(tm5005) C*. *elegans* animals raised at 20°C and 25°C throughout the reproductive period. In *fahd-1(tm5005) C*. *elegans* the total brood size is reduced compared to wild-type controls when raised at 20°C (left panel; 281 ±SD 28.11 for wt vs. 238 ±SD 63.96 for *fahd-1(tm5005)*; p = 0.05; N = 9) and even more severe when raised at 25°C (right panel; 199 ±SD 71.30 for wt vs. 98.8 ±SD 49.23 for *fahd-1(tm5005)*; p = 0.0013; N = 10). (D) Egg laying patterns were determined for wild-type and *fahd-1(tm5005) C*. *elegans*. *Left panel*: 4-day old wild-type and *fahd-1(tm5005)* animals were kept in M9 buffer (without bacteria) for four hours and the number of eggs that were laid by each genotype during this time was counted. While wild-type worms do not lay their eggs under these unfavorable conditions, *fahd-1(tm5005)* worms ‘lose’ them over the course of time (2.08 ±SD 1.75 for wt vs. 10.26 ±SD 0.32; p = 0.0598 for 60 min, p < 0.002 in all other cases; N = 3 experiments (72 worms total)). *Right panel*: Micrographs were prepared from 4-day old, well-fed and unstressed hermaphrodites of the wild-type and *fahd-1(tm5005)* genotype. While wild-type worms carry between 18 and 20 eggs, *fahd-1(tm5005)* animals show great variety in the number of eggs, tending to accumulate them in the uterus. Representative pictures are shown.

To address the function of FAHD-1 in worms, a deletion mutant, referred to as *fahd-1(tm5005)*, was generated and analyzed. First, DNA and protein lysates were prepared and analyzed to verify the mutant genotype. As shown by single worm duplex PCR, all worms of a randomly picked sample had the correct gene deletion, and successful disruption of the *fahd-1* gene was supported by results of Western blot, using a peptide-specific antibody recognizing specifically *C*. *elegans* FAHD-1 (Fig 3A). Together, these data suggest that FAHD-1 function is completely abrogated in the mutant animals.

The deletion mutant differed from the wild-type by a significant reduction in body size (S1 Fig) and a significant reduction in brood size (Fig 3B). When the egg laying behavior was further analyzed, we found that *fahd-1(tm5005)* animals at the young adult stage carried an irregular number of eggs (varying in number between 12 and 63) at the age of 96 hours. Under these conditions the number of eggs in wild-type worms was rather constant (i.e. between 18 and 20 eggs) (Fig 3C). When kept under starvation conditions (M9 media w/o bacteria), egg laying was inhibited in wild-type worms; under these conditions, *fahd-1(tm5005)* mutants continued to lay eggs, indicating that deletion of *fahd-1* causes an abnormality in the egg-laying process (Fig 3D), which may reflect muscular and/or neuronal changes in the egg laying apparatus (see also below, discussion).

### Inactivation of *fahd-1* leads to mitochondrial dysfunction and locomotion deficits

**Fig 4:**
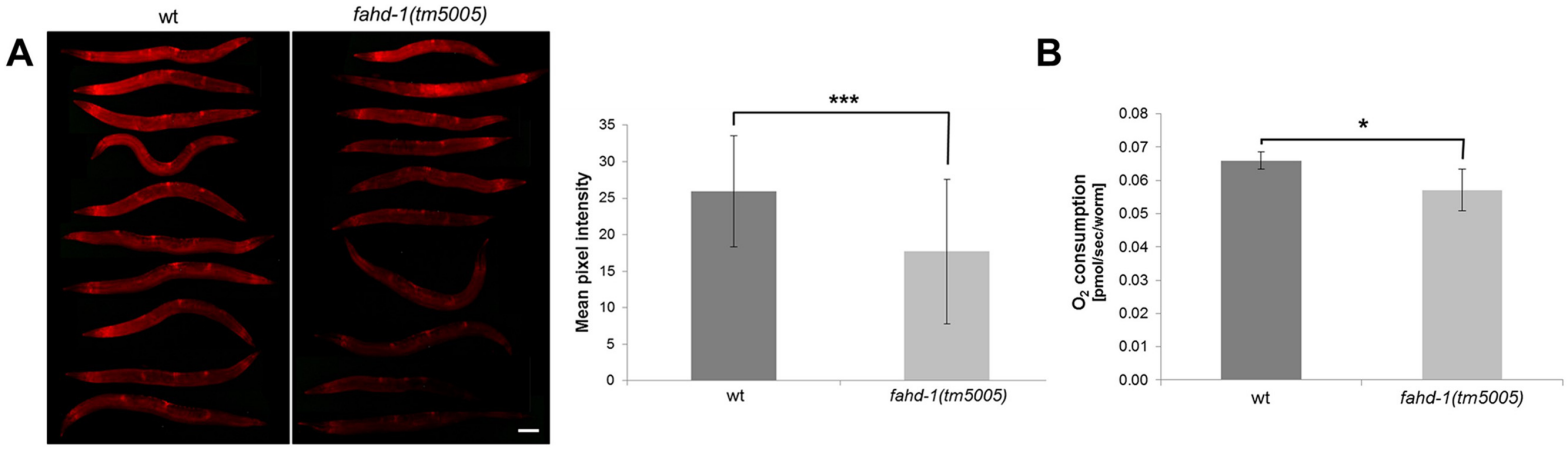
Mitochondrial dysfunction in *fahd-1(tm5005) C*. *elegans*. (A) 3-day old *fahd-1(tm5005)* and wild-type *C*. *elegans* were incubated with 100 nM TMRE overnight. *Left panel*: *fahd-1(tm5005)* animals exhibit a decreased mitochondrial membrane potential as shown by fluorescence microscopy. Representative images are shown. Size bar = 100 μm. *Right panel*: For quantification of the pixel intensities, fluorescence pictures were analyzed using ImageJ (25.93 ±SD 7.61 for wt vs. 17.69 ±SD 9.87 for *fahd-1(tm5005)*; p = 0.0005; N = 30). (B) Oxygen consumption rate was determined in wild-type and *fahd-1(tm5005)* animals by Oxygraph 2K measurement and the basic respiration rate was determined. In *fahd-1(tm5005)* animals this parameter is significantly reduced (0.0659 ±SD 0.0026 pmol sec^-1^ worm^-1^ for wt vs. 0.0.0571 ±SD 0.0062 pmol sec^-1^ worm^-1^ for *fahd-1(tm5005)*; p = 0.0475; N = 4).

Since oxaloacetate decarboxylase converts oxaloacetate, a key metabolite of the tricarboxylic acid (TCA) cycle, back into pyruvate, it is conceivable that mitochondrial function may be altered in *fahd-1(tm5005)* mutants. This was addressed by monitoring the mitochondrial membrane potential using the potential sensitive dye TMRE. Confocal microscopy of TMRE stained worms revealed intense and specific staining of discrete globular structures, consistent with discrete and potential-specific mitochondrial staining (S2 Fig). Whereas the overall membrane potential varied considerably between individual worms of either genotype, the average mitochondrial membrane potential was significantly reduced in *fahd-1(tm5005)* mutants (Fig 4A), suggesting that the absence of oxaloacetate decarboxylase activity reduces mitochondrial function. In support of this conclusion, we found that oxygen consumption of *fahd-1(tm5005)* mutants was significantly decreased compared to wild-type animals (Fig 4B).

**Fig 5:**
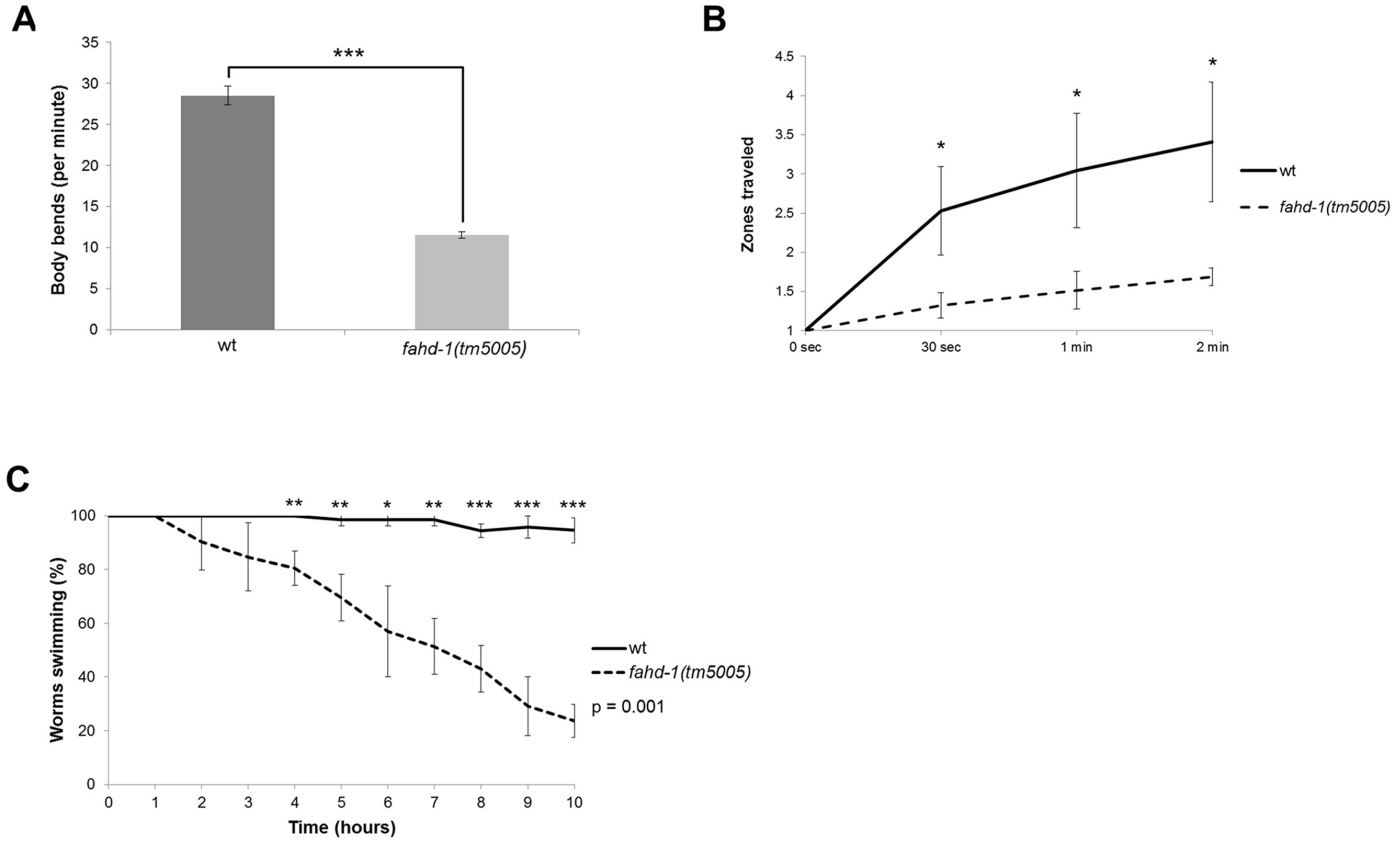
*fahd-1(tm5005) C*. *elegans* have a locomotion deficit. (A) 4-day old wild-type and *fahd-1(tm5005)* animals were placed on an empty NGM plate (without bacterial lawn) and the number of body bends was counted for one minute. *fahd-1(tm5005)* animals show fewer and more uncoordinated body bends compared to wild-type animals (28.53 ±SD 1.12 for wt vs. 11.57 ±SD 0.42 for *fahd-1(tm5005)*; p = 1.60E-05; N = 3 experiments (30 worms total)). (B) 4-day old wild type and *fahd-1(tm5005)* worms were put in the center of an NGM plate seeded with *E*. *coli* OP50. The radial dispersal rate (see Materials and Methods) was determined for three different time points (30 sec, 1 min, 2 min). The radial dispersal rate is decreased in *fahd-1(tm5005) C*. *elegans* compared to wild-type controls (3.41 ±SD 0.76 zones after two minutes for wt vs. 1.69 ±SD 0.11 zones after two minutes for *fahd-1(tm5005)*; p < 0.03 in all cases; N = 3 experiments (90 worms total)). (C) 4-day old wild-type and *fahd-1(tm5005)* hermaphrodites were screened for their swimming endurance performance in M9 buffer for up to 10 h. Wild-type worms are able to swim significantly longer than *fahd-1(tm5005)* worms (94.56% ±SD 4.72% swimming after 5 hours for wild-type vs. 23.72% ±SD 6.18 swimming after 10 hours for *fahd-1(tm5005)*; p = 0.001; N = 3 experiments (72 worms total)).

These findings prompted us to analyze locomotion behavior, which conceivably requires robust mitochondrial function. We found that the mobility of the worms, measured in an exploratory test, was significantly reduced for the *fahd-1(tm5005)* mutant (Fig 5A). Furthermore, the analysis of the radial dispersal rate revealed a significantly reduced locomotion during a 2-minute test period (Fig 5B). To address the capability of the worms for endurance physical activity, worms were subjected to a swimming assay in liquid medium, where the number of worms remaining actively swimming in liquid culture was monitored over a period of ten hours. In this assay, the *fahd-1(tm5005)* mutants displayed a significantly reduced physical performance (Fig 5C). The altered locomotion behavior of the *fahd-1(tm5005)* mutant strain was also evident when movement of the worms was monitored (S1 and S2 Movies). To verify that these phenotypes are not caused by second-site mutations introduced during the generation of the deletion mutant and not eliminated during outcrossing, a *fahd-1* rescue line was produced, in which the *fahd-1* gene (including promoter, exons, introns, and 3’-UTR) was transgenically expressed in *fahd-1* mutant worms. The mobility phenotypes of *fahd-1* worms could be partially rescued by the transgene (S3 Fig).

### Lifespan extension of mutant strain *fahd-1(tm5005)* is independent of UPR^mt^

**Fig 6:**
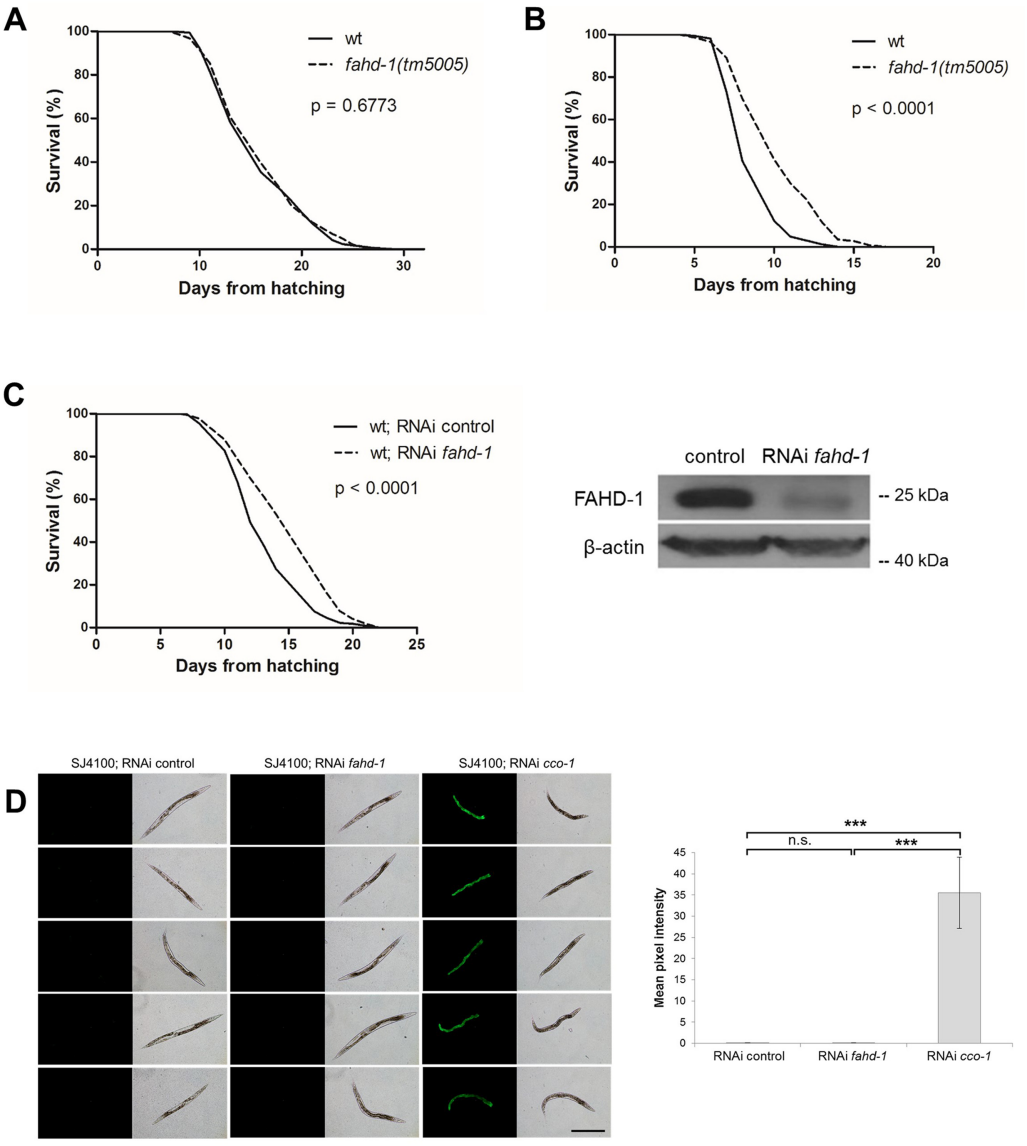
Lifespan of *fahd-1(tm5005)* animals is increased at elevated temperature. (A) Lifespan analysis of *fahd-1(tm5005) C*. *elegans* compared to wild-type *C*. *elegans* maintained at 20°C. There is no difference in the lifespans between the two strains (median survival: 16 days for both groups; p = 0.6773; N > 200). (B) Lifespan analysis of *fahd-1(tm5005) C*. *elegans* compared to wild-type *C*. *elegans* raised at 25°C. The lifespan of *fahd-1(tm5005) C*. *elegans* is significantly increased (median survival: 8 days for wt vs. 10 days for *fahd-1(tm5005)*; p < 0.0001; N > 200). (C) *Left panel*: FAHD-1 knock-down by ingested RNAi in wild-type worms also leads to an extension of the median lifespan when performed at 25°C (median survival: 12 days for RNAi control vs. 15 days for RNAi *fahd-1*; p < 0.0001; N > 200). *Right panel*: The efficiency of the knock-down was tested by Western blot using a peptide-specific rabbit polyclonal antibody against FAHD-1. β-actin served as a loading control. The lifespan assays were performed at least three times. One exemplary experiment is shown. (D) Representative fluorescence images (left panel) and quantitation of fluorescence intensities (right panel) of 3-day old worms of the strain SJ4100: N2;*Is*[p_*hsp-6*_GFP] that were fed with control bacteria or bacteria expressing dsRNA directed against *fahd-1* or *cco-1*. FAHD-1 knockdown by ingested RNAi does not activate the UPR^mt^ at 25°C. In control experiments, UPR^mt^ was induced by knock-down of *cco-1*. Size bar = 500 μm.

It is known that mild mitochondrial dysfunction, e.g. due to alterations in the stoichiometry of electron transport chain (ETC) complexes, can extend the lifespan of so-called “Mit” mutants of *C*. *elegans* [17]. We found that lifespan of the *fahd-1(tm5005)* mutant was unaltered with respect to the wild-type worms when assayed at 20°C (Fig 6A). When lifespan was analyzed at 25°C, we found that under these conditions the medium lifespan of *fahd-1(tm5005)* mutants was significantly increased (Fig 6B). Lifespan extension at 25°C could also be confirmed by RNA interference (Fig 6C).

In long-lived Mit mutants, lifespan extension is often mediated by activation of the mitochondrial unfolded protein response (UPR^mt^) [18]. To test if UPR^mt^ contributes to the lifespan extension of FAHD-1 depleted worms observed at 25°C, a UPR^mt^ reporter strain was used carrying a reporter construct in which the heat shock protein-6 (HSP-6) promoter drives expression of green fluorescence protein (GFP). As a positive control, ETC subunit *cco-1* was specifically depleted by RNA interference. This led to a strong positive GFP fluorescence signal in the treated worms, reflecting activation of UPR^mt^ [19]. Under these conditions, knocking down *fahd-1* by RNAi had no effect at either 25°C (Fig 6D) or 20°C (S4 Fig). Together these observations indicate that lifespan extension by knock-down of *fahd-1* does not involve activation of the UPR^mt^.

## Discussion

In the present communication, we have probed the function of *fahd-1*, the nematode homolog of the recently identified human oxaloacetate decarboxylase FAHD1, in the context of an intact organism. We found that mutation of *fahd-1* resulted in reduced brood size, a deregulation of the egg laying process and a severe locomotion deficit, characterized by reduced exploratory movements and reduced performance in an endurance exercise test. Notably, mitochondrial function was altered in the *fahd-1(tm5005)* mutant strain, as shown by a reduction of mitochondrial membrane potential and a reduced rate of oxygen consumption. Mitochondrial dysfunction was accompanied by lifespan extension in worms grown at elevated temperature; however, unlike in mutant worms with a defect in the electron transport chain, UPR^mt^ was not upregulated in worms upon inactivation of *fahd-1* by RNA interference. Together these data establish a role of *fahd-1* to maintain mitochondrial function and physical activity in nematodes.

Similar to the findings reported here for *fahd-1(tm5005)* animals, reduced physical fitness and an increased lifespan was observed in several nematode strains with genetic defects in subunits of individual complexes of the electron transport chain (ETC) or in other components modulating ETC substrate availability, generally referred to as “Mit” mutants [20]. However, the mutant nematodes generated here do not display defects in the ETC; instead, deletion of *fahd-1*, coding for a mitochondrial metabolic enzyme, seems to alter mitochondrial metabolism in a different way, conceivably by antagonizing pyruvate carboxylase (PC) which, under certain metabolic conditions, restores oxaloacetate levels through the carboxylation of pyruvate [21]. Whereas in many “Mit” mutants, disturbed stoichiometry of a given ETC complex leads to massive accumulation of unfolded proteins in the mitochondrial matrix, we anticipate that the ETC is intact in *fahd-1(tm5005)* animals. Consistent with this conclusion, UPR^mt^ was not activated in an UPR^mt^ reporter strain upon depletion of *fahd-1*.

The *fahd-1(tm5005)* mutant nematodes described here displayed some behavioral differences to the wild-type which may shed further light on the physiological function of *fahd-1* in nematodes. Besides an overall reduction in brood size, the mutant worms also displayed differences in egg-laying behavior. On the one hand, *fahd-1(tm5005)* animals started egg-laying slightly later than wild-type animals (data not shown) and, in contrast to the stereotypic egg-laying pattern displayed by wild-type worms, we observed a large variation in the number of eggs contained in *fahd-1(tm5005)* animals at any given time point. Moreover, the response of egg-laying to starvation [22] was disturbed in *fahd-1(tm5005)* animals. When deprived of food, egg-laying ceases in wild-type worms and fertilized eggs are retained in the uterus where they continue to develop. This modulation of egg-laying behavior by food involves an antagonistic action between serotonin (5HT) and dopamine: application of 5HT to worms immediately elicits egg laying in the absence of food [23,24], and dopamine inhibits egg laying induced by either 5HT or food [25]. Whereas wild-type worms did not lay any eggs under starvation conditions, *fahd-1(tm5005)* animals continued egg-laying under these conditions. In wild-type worms, egg-laying is an active process regulated by neuronal signals mediated by 5HT and other signals and requires an intact vulvar musculature. Accordingly, the changes in egg-laying behavior observed in *fahd-1(tm5005)* animals point to altered (reduced) function of the vulvar musculature, altered neuronal function or a combination of both. The same holds true for the altered changes in locomotion behavior, which may be due to altered (reduced) function of the body wall muscles and/or altered function of the corresponding neurons. Reduced muscular function of *fahd-1(tm5005)* animals may be explained by the observed reduction in mitochondrial activity.

Both the overall structure and all critical amino acid residues (in particular H30, E33, D102, R106; see [5]) known to be relevant for the ODx activity of human FAHD1 are conserved in the nematode FAHD-1 protein. Whereas an increase in oxaloacetate concentration was observed in both the kidney and liver of FAHD1^-/-^ mice [5], technical difficulties precluded the reproducible determination of oxaloacetate levels in worm lysates (A. Taferner *et al.*, unpublished). Thus, it remains to be formally established that nematode FAHD-1 protein decarboxylates oxaloacetate *in vivo*. If so, the inhibition of mitochondrial activity observed in *fahd-1(tm5005)* animals may be explained by increased intracellular concentration of oxaloacetate, which is known to inhibit several mitochondrial enzymes such as succinate dehydrogenase [26,27]. On the other hand, metabolites of the tricarboxylic acid cycle are well known precursors for essential neurotransmitters, such as glutamate and serotonin, and the sustained synthesis of such neurotransmitters requires the anaplerotic function of PC, i.e. the ability to produce oxaloacetate from (glycolytic) pyruvate. Since FAHD1 functions as antagonist of PC, at least in mice [5,21], it is tempting to speculate that any alterations of neuronal function in *fahd-1(tm5005)* animals could be related to the influence of FAHD-1 on the metabolism of relevant neurotransmitters, such as glutamate and serotonin. Whereas further work will be required to address these questions, the work reported here describes for the first time a functional analysis of FAHD-1 in an intact organism.

## Supporting Information

S1 Fig *fahd-1(tm5005) C*. *elegans* have a slightly reduced body size. (PDF)

S2 Fig The fluorescent dye TMRE specifically stains mitochondria dependent on mitochondrial membrane potential. (PDF)

S3 Fig The mobility defect of *fahd-1(tm5005) C*. *elegans* can be partly rescued. (PDF)

S4 Fig UPR^mt^ is not upregulated in FAHD-1 depleted worms cultivated at 20°C. (PDF)

S1 Movie Locomotion behavior of *fahd-1(tm 5005)* mutants. (AVI)

S2 Movie Locomotion behavior of wild type nematodes. Videos showing locomotion of *fahd-1(tm 5005)* mutants compared to wild-type nematodes. Animals were placed on an NGM agar plate without bacteria. Assays were performed at 20°C.
(AVI)

S1 Text Supplementary methods. Generation of fahd-1 rescue line & determination of body size
(PDF)
