# Supplementary material for: FAH Domain Containing Protein 1 (FAHD-1) Is Required for Mitochondrial Function and Locomotion Activity in C. elegans
Source: PLoS One. 2015 Aug 12;10(8):e0134161. doi: 10.1371/journal.pone.0134161 (PMC4534308; doi:10.1371/journal.pone.0134161)
Supplement: S1 Fig — (PDF) [file pone.0134161.s001.pdf]

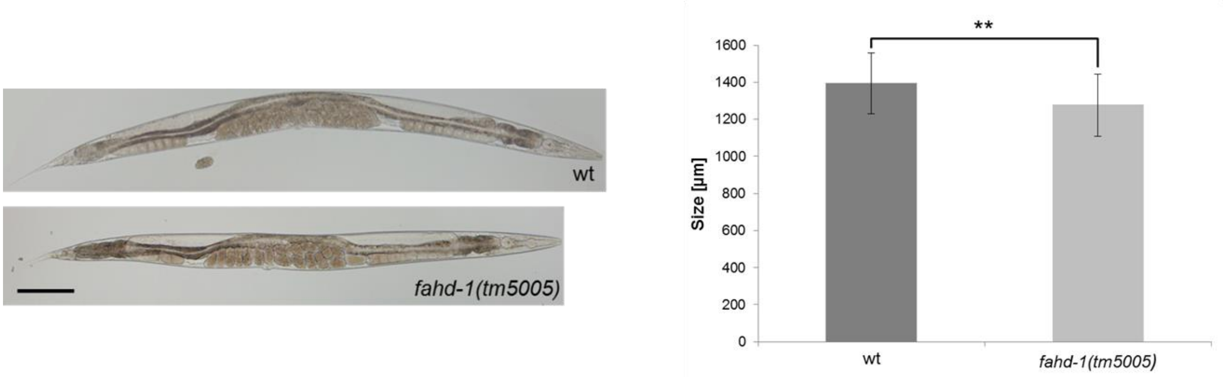

Supplementary Fig. 1: *fahd-1(tm5005) C. elegans* have a slightly reduced body size

4-day old gravid *fahd-1(tm5005)* and wild-type hermaphrodites were imaged (representative images shown in left panel) and their body size was determined (quantification shown on right panel). *fahd-1(tm5005) C. elegans* are slightly but significantly shorter compared to wild-type controls of the same age (1,395.14 ± SD 164.41 µm for wt vs. 1,277.56 ± SD 167.67 µm for *fahd-1(tm5005)*; p = 0.0031; N = 20). Scale bar: 100 µm
