# Supplementary material for: FAH Domain Containing Protein 1 (FAHD-1) Is Required for Mitochondrial Function and Locomotion Activity in C. elegans
Source: PLoS One. 2015 Aug 12;10(8):e0134161. doi: 10.1371/journal.pone.0134161 (PMC4534308; doi:10.1371/journal.pone.0134161)
Supplement: S2 Fig — (PDF) [file pone.0134161.s002.pdf]

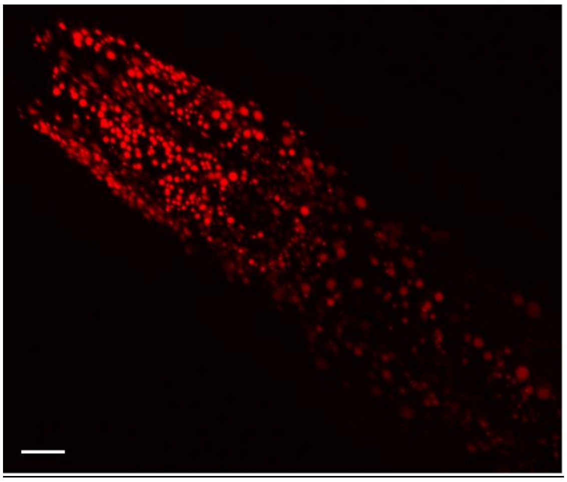

Supplementary Fig. 2: The fluorescent dye TMRE specifically stains mitochondria dependent on mitochondrial membrane potential

Confocal images (Z-stack projection) of the pharynx region of a 3-day old wild-type animal stained overnight with the membrane potential-sensitive mitochondrial dye tetramethylrhodamine ethyl ester (TMRE). Scale bar: 10 µm
