# Supplementary material for: FAH Domain Containing Protein 1 (FAHD-1) Is Required for Mitochondrial Function and Locomotion Activity in C. elegans
Source: PLoS One. 2015 Aug 12;10(8):e0134161. doi: 10.1371/journal.pone.0134161 (PMC4534308; doi:10.1371/journal.pone.0134161)
Supplement: S3 Fig — (PDF) [file pone.0134161.s003.pdf]

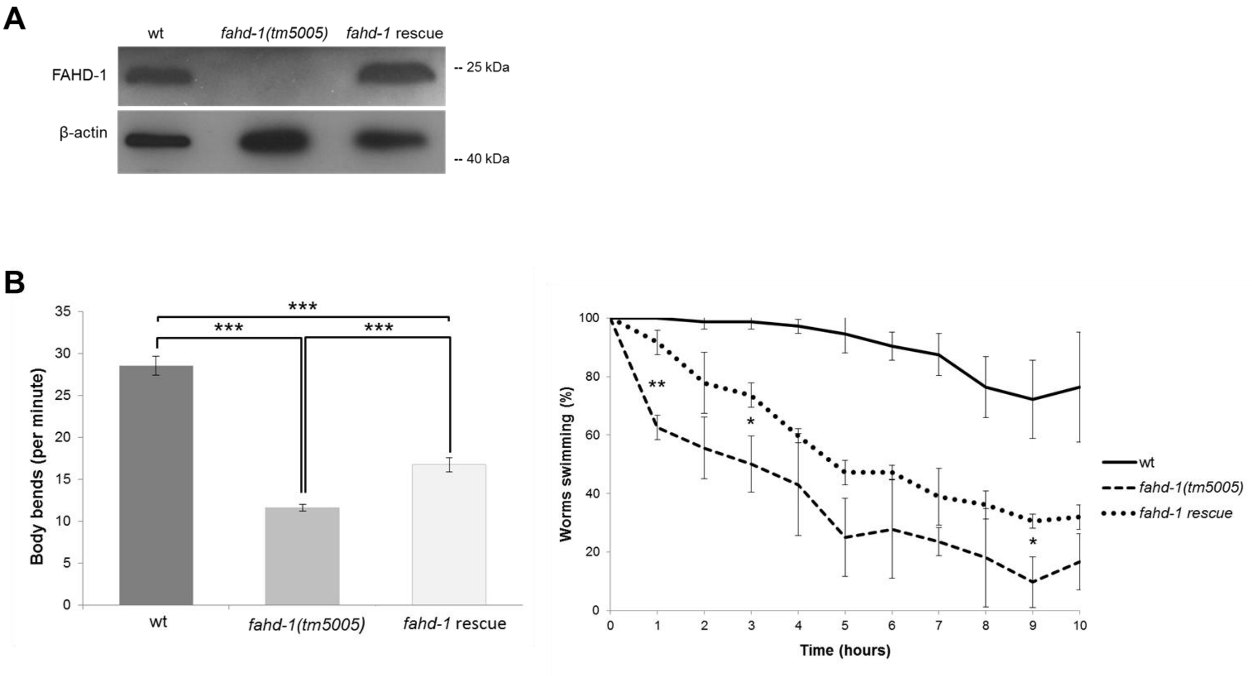

Supplementary Fig. 3: The mobility defect of *fahd-1(tm5005) C. elegans* can be partly rescued

(A) The presence of the FAHD-1 protein in *fahd-1;Ex*[$\text{p}_{fahd\text{-}1}$FAHD-1; L4040] transgenic animals was shown by Western blot with a peptide-specific antibody against FAHD-1. β-actin served as a loading control. (B) *fahd-1;Ex*[$\text{p}_{fahd\text{-}1}$FAHD-1; L4040] animals performed better than *fahd-1(tm5005)* mutants in the exploratory test (left panel; wt: 28.53 ±SD 1.12; *fahd-1(tm5005):* 11.57 ±SD 0.42; rescue: 16.73 ±SD 0.85; N = 3 experiments (30 worms total)) and in the swimming assay (right panel; N = 3 experiments (72 worms total)).
