# Supplementary material for: FAH Domain Containing Protein 1 (FAHD-1) Is Required for Mitochondrial Function and Locomotion Activity in C. elegans
Source: PLoS One. 2015 Aug 12;10(8):e0134161. doi: 10.1371/journal.pone.0134161 (PMC4534308; doi:10.1371/journal.pone.0134161)
Supplement: S4 Fig — (PDF) [file pone.0134161.s004.pdf]

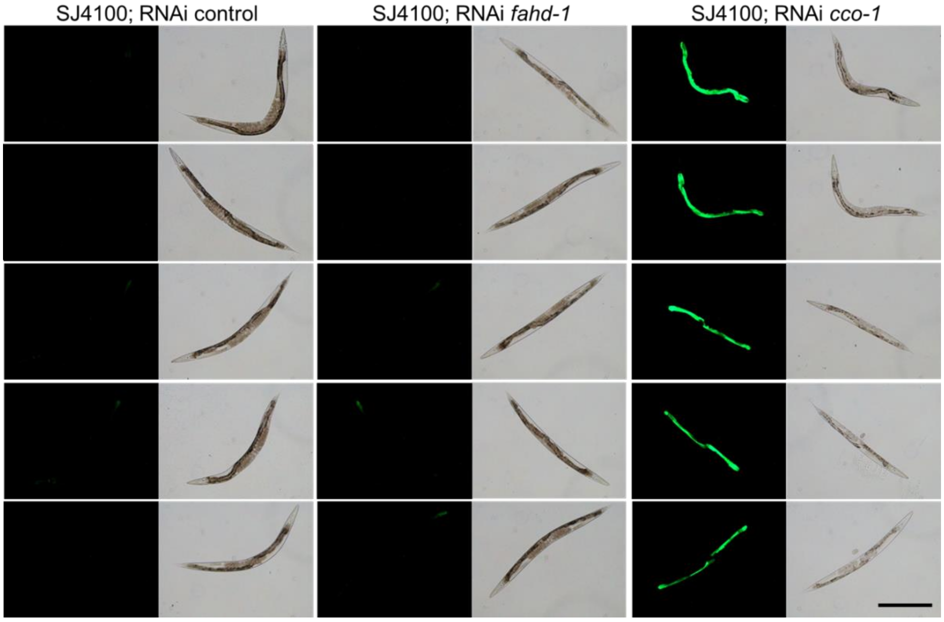

Supplementary Fig. 4: UPR^{mt} is not upregulated in FAHD-1 depleted worms cultivated at 20 °C

Representative images of 3-day old worms of the strain SJ4100: N2;*Is*[p$_{hsp-6}$GFP] that were fed with control bacteria or bacteria expressing dsRNA directed against *fahd-1* or *cco-1*. FAHD-1 knockdown by ingested RNAi does not activate the UPR^{mt} at 20 °C. In control experiments, UPR^{mt} was induced by knock-down of *cco-1*. Size bar = 500 µm.
