# Supplementary material for: FAH Domain Containing Protein 1 (FAHD-1) Is Required for Mitochondrial Function and Locomotion Activity in C. elegans
Source: PLoS One. 2015 Aug 12;10(8):e0134161. doi: 10.1371/journal.pone.0134161 (PMC4534308; doi:10.1371/journal.pone.0134161)
Supplement: S1 Text — Generation of fahd-1 rescue line & determination of body size (PDF) [file pone.0134161.s007.pdf]

Supplementary methods

Generation of *fahd-1* rescue line

For the rescue line *fahd-1;Ex*[p$_{fahd\text{-}1}$FAHD-1; L4040], the *fahd-1* gene (including promoter region, exons, introns, and 3'-UTR) was cloned into the pCRII® TOPO® vector (Invitrogen, Carlsbad, USA) by TA cloning. The insert was amplified from genomic DNA using the primers ATATCAGGTTCCTCATACCAGG (forward primer) and ATTTGTGGAGAACTCTGCAAAAATC (reverse primer). The construct was injected into the cytoplasm of the syncytial gonad of young adult N2 ancestral *C. elegans* following standard protocols. For identification of transformants, L4040 (gift from Andrew Fire; Addgene plasmid #1621), which leads to expression of GFP in the pharynx, was co-injected. The F1 offspring was observed for evidence of the transgenic marker and each F1 transformant was separately cloned onto a new plate. F2 animals that had inherited and expressed the transgenic array were considered as stable lines.

Determination of body size

To determine body size, pictures of 4-day old worms were taken and their total length was measured applying a curved line from mouth to tail. The measurement was performed on 20 individual worms of each strain using the camera software ProgRes® CapturePro (Version 2.8.8; Jenoptic Optical Systems, Jena, Germany). The significance level was determined using the Student's t-test.
